# Prognostic significance of a combined and controlled nutritional status score and EBV-DNA in patients with advanced nasopharyngeal carcinoma: a long-term follow-up study

**DOI:** 10.20892/j.issn.2095-3941.2020.0627

**Published:** 2021-06-16

**Authors:** Hui Lu, Shanshan Guo, Liting Liu, Qiuyan Chen, Yujing Liang, Sailan Liu, Xuesong Sun, Qingnan Tang, Xiaoyun Li, Ling Guo, Haoyuan Mo, Linquan Tang, Haiqiang Mai

**Affiliations:** 1 State Key Laboratory of Oncology in South China; Collaborative Innovation Center for Cancer Medicine; Guangdong Key Laboratory of Nasopharyngeal Carcinoma Diagnosis and Therapy, Sun Yat-sen University Cancer Center, Guangzhou 510060, China; 2 Department of Radiation Oncology, Guangzhou Concord Cancer Center, Guangzhou 510045, China; 3 Department of Nasopharyngeal Carcinoma, Sun Yat-sen University Cancer Center, Guangzhou 510060, China

**Keywords:** Controlling nutritional status score, Epstein-Barr virus deoxyribonucleic acid, nasopharyngeal carcinoma, prognosis, predictive factor

## Abstract

**Objective:**

Several studies have reported that the controlling nutritional status (CONUT) score is a prognostic predictor for survival among patients with different types of cancer. We assessed the prognostic value of changes in the CONUT score during treatment and the ΔCONUT-EBV DNA score in patients with advanced nasopharyngeal carcinoma (NPC).

**Methods:**

We retrospectively analyzed 433 patients with advanced NPC having no evidence of metastasis from January 2007 to June 2011; the patients underwent radical concurrent chemoradiotherapy (CCRT) at Sun Yat-sen University Cancer Center and were grouped based on their ΔCONUT and ΔCONUT-EBV DNA scores. Kaplan-Meier curves were used to compare the patient outcomes according to the cut-off ΔCONUT score and the ΔCONUT-EBV DNA scoring system.

**Results:**

Among all patients, overall survival (OS) was independently predicted by a high ΔCONUT score (*P* = 0.031) and high EBV DNA (*P* < 0.001). The ΔCONUT-EBV DNA score [OS area under the curve (AUC) = 0.621; progression free survival (PFS)-AUC = 0.612; distant metastasis-free survival (DMFS)-AUC = 0.622] was more predictive of OS, PFS, and DMFS in patients with advanced NPC than the ΔCONUT score (OS-AUC = 0.547; PFS-AUC = 0.533; DMFS-AUC = 0.522) and pretreatment plasma EBV DNA levels alone (OS-AUC = 0.600; PFS-AUC = 0.591, DMFS-AUC = 0.610). The ΔCONUT-EBV DNA score was significantly correlated with OS, PFS, and DMFS in patients with advanced NPC treated with CCRT.

**Conclusions:**

The ΔCONUT-EBV DNA score may be useful in clinical practice as a convenient biomarker for predicting the outcomes in patients with advanced NPC treated with CCRT.

## Introduction

Nasopharyngeal carcinoma (NPC) is one of the most prevalent malignancies in Southeast Asia, including southern China^1^. Epstein-Barr virus deoxyribonucleic acid (EBV DNA) has been used as a plasma marker for population screening^2^, prognoses^3^, and disease recurrence surveillance^4,5^. Owing to the high sensitivity of irradiation, radiotherapy alone and concurrent chemoradiotherapy (CCRT) are the primary treatments for nonmetastatic NPC. Moreover, intensity-modulated radiotherapy (IMRT) is the currently preferred method, because it reduces the risk of short-term side effects and long-term sequelae^6^. Over the years, immune-nutritional scores, such as the body mass index (BMI)^7^, albumin-globulin ratio^8^, and prognostic nutritional index (PNI)^9,10^, have been reported to be prognostic markers that influence NPC treatment outcomes.

Recently, studies have shown that the controlling nutritional status (CONUT) score, a novel immunological and nutritional score, is a prognostic predictor for survival prognosis in many cancer types^11–16^. This score is calculated from the total lymphocyte count, total cholesterol, and serum albumin levels^17^. However, data on the prognostic value of the CONUT score for patients with advanced NPC are still unavailable. Moreover, the point at which the nutritional score should be measured during anti-cancer treatment remains unclear. Thus, this study aimed to clarify the prognostic and predictive values of the CONUT score at different time points to predict survival among patients with advanced NPC.

However, nutritional scores, such as the CONUT score, PNI, and BMI, are not always reliable in predicting the outcomes of cancers, because they lack tumor-related factors. Previous reports have suggested that tumor-related factors, such as the EBV DNA level^3^ and tumor node metastasis (TNM) stage^18,19^, are likely the most reliable prognostic predictors for NPC. Plasma marker EBV DNA has been used for screening^2^, prognoses^3^, and disease recurrence surveillance^4,5^. Lin et al.^20^ reported an inferior overall survival (OS) in patients with higher baseline plasma EBV DNA levels. The addition of pretreatment plasma EBV DNA to the 8^th^ edition of the AJCC/UICC TNM stage classification greatly improved its prognostic performance^21–23^. Many studies support a positive relationship between the plasma EBV DNA level and tumor burden^24,25^. Unfortunately, cut-off values of plasma EBV DNA levels varied across these studies, and an optimal cut-off value of this liquid biopsy marker is still being determined^26,27^. Here, we characterized the complementary role of the ΔCONUT score in combination with pretreatment plasma EBV DNA. The ΔCONUT-EBV DNA score, which is based on the ΔCONUT score and pretreatment plasma EBV DNA, was integrated with nutritional score and tumor-related factors, so it may have more prognostic information compared to only 1 parameter.

## Materials and methods

### Patients and study design

We retrospectively included and analyzed 433 patients with advanced NPC, but with no evidence of metastasis (M0) from January 2007 to June 2011, who underwent radical CCRT at Sun Yat-sen University Cancer Center. The exclusion and inclusion criteria were as follows: (I) patients with newly-diagnosed and pathologically proven NPC; (II) patients with complete clinical information and follow-up data; (III) patients with complete laboratory data; (IV) patients who underwent radical CCRT during the course of anti-cancer treatment; (V) patients without other malignant cancers, serious illnesses (severe acute or chronic diseases); (VI) patients without distant metastasis; and (VII) patients with confirmed locoregionally advanced (stages II–IVA) NPC, as defined by the 2010 Union for International Cancer Control (UICC) system (**Supplementary Figure S1**). Laboratory data and clinical and epidemiological characteristics of the patients, such as age, gender, date of diagnosis, UICC T stage, UICC N stage, clinical stage, total lymphocyte count (/mm^3^), total cholesterol (mg/dL) and serum albumin (g/dL) levels, were assessed from their medical records. All patients were followed-up until April 2020 or until death. Our study protocol was approved by the ethics committee of Sun Yat-sen University Cancer Center (Approval No. GZR2014-069).

### Treatment strategies

Institutional guidelines recommend CCRT ± neoadjuvant/adjuvant chemotherapy (CCRT ± NC/AC) for patients with stage II–IVA NPC. In the present study, 397 (91.7%) of the patients received CCRT only, 24 (5.5%) received CCRT + AC, and 12 (2.8%) received CCRT + NC. All patients received IMRT. The prescribed doses were 66–70 Gy to the primary tumor and 54–64 Gy to the involved cervical lymph nodes and low risk clinical target volume.

### The CONUT score and cut-off value

The CONUT score was calculated based on the total cholesterol level, total lymphocyte counts, and serum albumin level (**Supplementary Table S1**)^17^. Pretreatment laboratory data (pre-CONUT score) were obtained within 14 days before anti-cancer treatment. Post-CONUT scores were calculated based on the results of blood tests within 5 days before the completion of CCRT. Individual difference value (ΔCONUT score) of the post-treatment to pretreatment CONUT score was calculated as follows: (post - CONUT score) - (pre-CONUT score). The area under the curve (AUC) estimation method was used to determine the predictive value of the pretreatment CONUT score, the post-treatment CONUT score, and the ΔCONUT score for OS. The receiver operating characteristic (ROC) curve analysis method was used to determine the optimal ΔCONUT score cut-off value that was significantly correlated with OS.

### Definition of the ΔCONUT-EBV DNA score

The cut-off value of the ΔCONUT score was 3, which was used as the criterion to divide the included 433 patients into ΔCONUT-low (score ≤ 3) and ΔCONUT-high (score > 3) groups. The median cut-off value of EBV DNA was considered in all patients. The patients were divided into the following 2 groups according to the cut-off value of pretreatment plasma EBV DNA level (2,110 copies/mL): EBV DNA-low (score ≤ 2,110 copies/mL) and EBV DNA-high (score > 2,110 copies/mL) groups. Based on the cut-off values of the ΔCONUT score and pretreatment plasma EBV DNA, the ΔCONUT-EBV DNA score was defined. Patients with an EBV DNA score of ≤2,110 copies/mL [*N* = 217; low-risk group (LRG)] were assigned a score of 1; those with both a ΔCONUT score of ≤ 3 and an EBV DNA score of > 2,110 copies/mL [*N* = 170; middle-risk group (MRG)] were assigned a score of 2; and those with both a ΔCONUT score of > 3 and an EBV DNA score of > 2,110 copies/mL [*N* = 46; high risk group (HRG)] were assigned a score of 3 (**Table 3**).

### Follow-up

All the patients were routinely followed up every 3–4 months throughout the first 2 years, every 6 months for the next 2 years, and annually thereafter, and were monitored either until April 2020 or until death. OS was defined as the period from the initial diagnosis to the patient’s death or last follow-up, regardless of whether it was or was not related to NPC. The median follow-up period was 9.52 (range, 8.88–10.74) years. The following end points (time to the first defining event) were assessed: OS, progression-free survival (PFS), and DMFS.

### Statistical analysis

Pathological and clinical characteristics, and laboratory data were compared between the 2 groups using Fisher’s exact test. Survival curves were estimated using the Kaplan-Meier method with the log-rank test. Univariate and multivariate analyses were performed using the Cox proportional hazards model. In all patients, the median cut-off values were considered for age and EBV DNA. Age (cut-off: 46 years), gender (female/male), UICC T stage (T1–2, T3–4), UICC N stage (N0–1, N2–3), clinical stage (stage II, stage III–IVA), EBV DNA score (cut-off: 2,110 copies/mL), and ΔCONUT score (cut-off, 3) were the parameters considered in the Cox proportional hazards model. To further investigate the characteristics of patients between the different ΔCONUT-EBV DNA score groups, we performed further experiments. Fisher’s exact test with a two-sided significance level was used to compare the pathological and clinical characteristics, and laboratory data between the different ΔCONUT-EBV DNA score groups; all characteristics are shown in **Table 1** before the Bonferroni-Holm correction was included. These analyses were also corrected for testing using the Bonferroni-Holm method. All data were analyzed using SPSS statistical software for Windows, version 22.0 (SPSS, Chicago, IL, USA). All *P* values were two-sided. *P* < 0.05 and Bonferroni-corrected *P* < 0.05 were considered statistically significant.

**Table 1:** Baseline characteristics of 433 patients with locoregionally advanced nasopharyngeal carcinoma

Characteristics	ΔCONUT > 3 *N* = 88 (%)	ΔCONUT ≤ 3 *N* = 345 (%)	*P*
Age (years)			1
≤46	47 (53.4)	183 (53.0)	
>46	41 (46.6)	162 (47.0)	
Gender			0.177
Female	18 (20.5)	96 (27.8)	
Male	70 (79.5)	249 (72.2)	
UICC T stage			0.886
1	6 (6.8)	17 (4.1)	
2	17 (19.3)	63 (18.3)	
3	49 (55.7)	202 (58.6)	
4	16 (18.2)	66 (19.1)	
UICC N stage			0.603
0	13 (14.8)	39 (11.3)	
1	32 (36.4)	151 (43.8)	
2	35 (39.8)	127 (36.8)	
3	8 (9.1)	28 (8.1)	
Clinical stage			0.957
II	8 (9.1)	28 (8.1)	
III	57 (64.8)	226 (65.5)	
IV	23 (26.1)	91 (26.4)	
WHO histology			0.518
II	4 (4.5)	11 (3.2)	
III	84 (95.5)	334 (96.8)	
Smoking status			0.928
Non-smoker	53 (60.2)	215 (62.3)	
Ex-smoker	6 (6.8)	21 (6.1)	
Current smoker	29 (33.0)	109 (31.6)	
EBV DNA (copies/mL)			0.635
≤2,110	42 (47.7)	175 (50.7)	
>2,110	46 (52.3)	170 (49.3)	
VCA/IgA titers			0.349
≤80	28 (31.8)	91 (26.4)	
>80	60 (68.2)	254 (73.6)	
EA/IgA titers			0.066
≤20	62 (70.5)	206 (59.7)	
>20	26 (29.5)	139 (40.3)	

CONUT, controlling nutritional status score; N stage, node stage; T stage, tumor stage; UICC, Union for International Cancer Control; WHO, World Health Organization; EBV DNA, Epstein-Barr virus deoxyribonucleic acid; VCA, viral capsid antigen; EA, early antigen; IgA, immunoglobulin a.

## Results

### Clinical and pathological characteristics, and treatment outcomes

A total of 397 (91.7%) patients received CCRT only, 24 (5.5%) received CCRT + AC, and 12 (2.8%) received CCRT + NC. All patients in this cohort received IMRT. Of the 433 patients, 39 (9.0%) developed locoregional recurrence, 75 (17.3%) developed distant metastases, and 98 (22.6%) died. The 5-year OS, PFS, and DMFS were 86.4%, 77.8%, and 84.2%, respectively.

**Figure 1:**
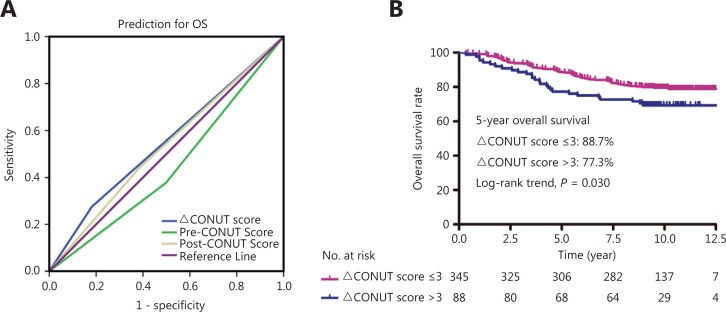
(A) Receiver operating characteristic (ROC) curve analysis of the pretreatment controlling nutritional status (CONUT), post-treatment CONUT, and ΔCONUT scores of interest for predicting overall survival (OS). (B) OS based on the ΔCONUT score.

We used the continuous variable pre-CONUT, post-CONUT, and ΔCONUT scores as the test variables, and OS as the state variable. The ΔCONUT score AUC was 0.547 [95% confidence interval (CI): 0.479–0.615], which was higher than the AUC of the other CONUT scores [pre-CONUT score (AUC = 0.453; 95% CI: 0.387–0.520) and post-CONUT score (AUC = 0.526; 95% CI: 0.458–0.594)] (**Figure 1A**). According to the ROC curve analysis results, the optimal ΔCONUT score cut-off value that significantly correlated with OS was 3 (AUC = 0.547; **Figure 1A**).

Based on the ΔCONUT score cut-off determined using the ROC curve analysis, the 433 patients were subdivided into the ΔCONUT-low (score ≤ 3; *N* = 345) and ΔCONUT-high (score > 3; *N* = 88) groups. The clinical and demographic characteristics of the patients in the 2 groups are shown in **Table 1**. Age, gender, cancer stage, histology, smoking status, EBV DNA levels, viral capsid antigen (VCA)/immunoglobulin a (IgA) titers, and early antigen (EA)/IgA titer distributions did not significantly differ between the 2 groups (**Table 1**).

**Figure 1B** shows the Kaplan-Meier curves for OS according to the ΔCONUT score-based groups. The patients in the ΔCONUT-high group were more likely to experience a shorter OS than those in the ΔCONUT-low group (**Figure 1B**; *P* = 0.030). The 5-year OS rates for patients with low and high ΔCONUT scores were 88.7% and 77.3%, respectively.

### Prognostic value of the ΔCONUT score

Univariate analyses identified an age > 46 years (*P* = 0.011), male (*P* = 0.008), advanced N stage (*P* = 0.003), high ΔCONUT score (*P* = 0.031), and high EBV DNA level (*P* < 0.001) as factors significantly associated with a worse OS (**Table 2**). Multivariable analyses showed that age [hazard ratio (HR): 1.580; 95.0% CI: 1.054–2.369; *P* = 0.027], gender (HR: 1.937; 95.0% CI: 1.115–3.367; *P* = 0.019), N stage (HR: 1.597; 95.0% CI: 1.060–2.406; *P* = 0.025), EBV DNA level (HR: 1.753; 95.0% CI: 1.142–2.693; *P* = 0.010), and ΔCONUT score (HR: 1.574; 95.0% CI: 1.009–2.454; *P* = 0.045) were independent prognostic factors for OS (**Table 2**).

**Table 2:** Univariate and multivariate analysis for OS

Variable	Univariate analysis		Multivariate analysis	
	HR (95% CI)	*P*	HR (95% CI)	*P*
Age	1.681 (1.125–2.512)	0.011	1.580 (1.054–2.369)	0.027
Gender	2.105 (1.214–3.648)	0.008	1.937 (1.115–3.367)	0.019
UICC T stage	0.922 (0.578–1.471)	0.734	0.796 (0.464–1.365)	0.955
UICC N stage	1.821 (1.219–2.720)	0.003	1.597 (1.060–2.406)	0.025
Clinical stage	3.077 (0.975–9.712)	0.055	2.591 (0.713–9.413)	0.214
ΔCONUT score	1.627 (1.045–2.535)	0.031	1.574 (1.009–2.454)	0.045
EBV DNA	2.116 (1.396–3.207)	<0.001	1.753 (1.142–2.693)	0.010

OS, overall survival; HR, hazard ratio; CI, confidence interval; N stage, node stage; T stage, tumor stage; UICC, Union for International Cancer Control; CONUT score, controlling nutritional status score; EBV DNA, Epstein-Barr virus deoxyribonucleic acid.

### Prognostic values of the ΔCONUT-EBV DNA score

**Figure 2:**
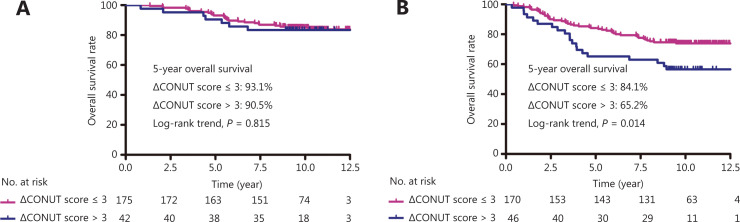
Survival curves of patients in different Epstein-Barr virus deoxyribonucleic acid (EBV DNA) groups. (A) Overall survival (OS) based on the ΔCONUT score in 217 patients with advanced nasopharyngeal carcinoma (NPC) with EBV DNA ≤ 2,110 copies/mL. (B) OS based on the ΔCONUT score in 216 patients with advanced NPC with EBV DNA > 2,110 copies/mL.

In addition, patients were stratified according to their pretreatment plasma EBV DNA levels. The results showed that the ΔCONUT score was not associated with OS in patients with low EBV DNA levels (≤2,110 copies/mL; **Figure 2A**). Notably, the high ΔCONUT score group had a significantly worse OS than the low ΔCONUT score group, but only for patients with high pretreatment plasma EBV DNA levels (>2,110 copies/mL; *P* = 0.014; **Figure 2B**).

We then suspected that the pretreatment plasma EBV DNA level could help improve the prognostic value of ΔCONUT in patients with advanced NPC. The ΔCONUT-EBV DNA score, a novel prognostic marker, was calculated based on the ΔCONUT score and the pretreatment plasma EBV DNA level (**Table 3**). Using this ΔCONUT-EBV DNA scoring system, we divided patients into low-risk (LRG; *N* = 217), middle-risk (MGR; *N* = 170), and high-risk (HRG; *N* = 46) groups (**Table 3**). The baseline characteristics of the different groups are shown in **Table 4**. Patients with a low ΔCONUT-EBV DNA score had earlier UICC N stages (Bonferroni-Holm corrected *P* = 0.003), earlier clinical stages (Bonferroni-Holm corrected *P* = 0.006), and lower EA/IgA values (Bonferroni-Holm corrected; *P* = 0.036) than patients with a medium ΔCONUT-EBV DNA score (MRG). Significant differences were present between LRG and HRG in the UICC N stage (Bonferroni-Holm corrected; *P* < 0.001), but other variables were not significantly different. There was no significant difference in the age, gender, stage, histology, smoking status, VCA/IgA titers, and EA/IgA titers between the HRG and MRG (**Table 4**).

**Table 3:** ΔCONUT-EBV DNA score based on risk stratification with CONUT scores and EBV DNA for advanced nasopharyngeal carcinoma patients

ΔCONUT-EBV group	ΔCONUT-EBV score	ΔCONUT score	EBV DNA
Low-risk group	1	–	≤2,110
Middle-risk group	2	≤3	>2,110
High-risk group	3	>3	>2,110

CONUT, controlling nutritional status score; EBV DNA, Epstein-Barr virus deoxyribonucleic acid.

**Table 4:** Characteristics of NPC patients in 3 different risk (ΔCONUT-EBV DNA score) groups

Characteristics	LRG *N* = 217 (%)	MRG *N* = 170 (%)	HRG *N* = 46 (%)	*P*			*Bonferroni holm* corrected *P*		
				LRG *vs.* MRG	LRG *vs.* HRG	MRG *vs.* HGR	LRG *vs.* MRG	LRG *vs.* HRG	MRG *vs.* HGR
Age (years)				0.033	0.184	1.000	0.099	0.552	1.000
≤46	127 (58.5)	81 (47.6)	22 (47.8)						
>46	90 (41.5)	89 (52.4)	24 (52.2)						
Gender				0.051	0.042	0.234	0.153	0.126	0.702
Female	65 (30.0)	42 (24.7)	7 (15.2)						
Male	152 (70.0)	128 (75.3)	39 (84.8)						
UICC T stage				0.248	0.702	0.162	0.744	1.000	0.486
1	12 (5.5)	4 (2.4)	4 (8.7)						
2	42 (19.4)	28 (16.5)	10 (21.7)						
3	127 (58.5)	101 (59.4)	23 (50.0)						
4	36 (16.8)	37 (21.8)	9 (19.6)						
UICC N stage				0.001	<0.001	0.334	0.003	<0.001	1.000
0	38 (17.5)	13 (7.6)	1 (2.2)						
1	101 (46.5)	65 (38.2)	17 (37.0)						
2	67 (30.9)	75 (44.1)	20 (43.5)						
3	11 (5.1)	17 (10.0)	8 (17.4)						
Clinical stage				0.002	0.119	0.263	0.006	0.357	0.789
II	26 (12.0)	6 (3.5)	4 (8.7)						
III	146 (67.3)	111 (65.3)	26 (56.5)						
IV	45 (20.7)	53 (31.2)	16 (34.8)						
WHO histology				1.000	0.704	0.679	1.000	1.000	1.000
II	7 (3.2)	6 (3.5)	2 (4.3)						
III	210 (96.8)	164 (96.5)	44 (95.7)						
Smoking status				0.440	0.127	0.451	1.000	0.381	1.000
Non-smoker	143 (65.9)	102 (60.0)	23 (50.0)						
Ex-smoker	13 (6.0)	10 (5.9)	4 (8.7)						
Current smoker	61 (28.1)	58 (34.1)	19 (41.3)						
VCA IgA				0.023	0.412	0.554	0.069	1.000	1.000
≤80	70 (32.3)	37 (21.8)	12 (26.1)						
>80	147 (67.7)	133 (78.2)	34 (73.9)						
EA IgA				0.012	0.940	0.131	0.036	1.000	0.393
≤20	145 (66.8)	92 (54.1)	31 (67.4)						
>20	72 (33.2)	78 (45.9)	15 (32.6)						

NPC, nasopharyngeal carcinoma; LRG, low-risk group = ΔCONUT-EBV DNA Score 1 (EBV DNA ≤ 2,110 copies/mL); MRG, middle-risk group = ΔCONUT-EBV DNA Score 2 (both ΔCONUT Score ≤ 3 and EBV DNA > 2,110 copies/mL); HRG, high-risk group = ΔCONUT-EBV DNA Score 3 (both ΔCONUT Score > 3 and EBV DNA > 2,110 copies/mL); CONUT, controlling nutritional status score; N stage, node stage; T stage, tumor stage; UICC, Union for International Cancer Control; WHO, World Health Organization; EBV DNA, Epstein-Barr virus deoxyribonucleic acid; VCA, viral capsid antigen; EA, early antigen; IgA, immunoglobulin a.

**Figure 3:**
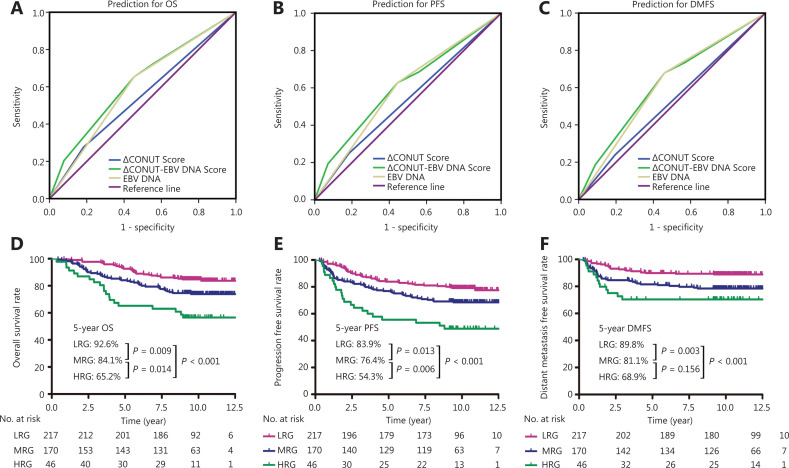
Receiver operating characteristic (ROC) curve analysis of the ΔCONUT score, the Epstein-Barr virus deoxyribonucleic acid (EBV DNA) level, and the ΔCONUT-EBV DNA score of interest for predicting 5-year overall survival (OS) (A), progression-free survival (PFS) (B), and distant metastasis-free survival (DMFS) (C); Survival analysis for 5-year OS (D), PFS (E), and DMFS (F) based on the ΔCONUT-EBV DNA score.

ROC analysis was used to evaluate the effect of the ΔCONUT score, pretreatment plasma EBV DNA level, and ΔCONUT-EBV DNA score on the prognosis. The results showed that the ΔCONUT-EBV DNA scores (OS: AUC = 0.621, 95% CI: 0.556–0.685; PFS: AUC = 0.612, 95% CI: 0.552–0.672; DMFS: AUC = 0.622, 95% CI: 0.552–0.672; **Figure 3A–3C**) were more predictive of OS, PFS, and DMFS in patients with advanced NPC than the ΔCONUT scores (OS: AUC = 0.547, 95% CI: 0.480–0.613; PFS: AUC = 0.533, 95% CI: 0.472–0.594; DMFS: AUC = 0.522, 95% CI: 0.449–0.595; **Figure 3A–3C**) or pretreatment plasma EBV DNA levels alone (OS: AUC = 0.600, 95% CI: 0.537–0.663; PFS: AUC = 0.591, 95% CI: 0.532–0.650; DMFS: AUC = 0.610, 95% CI: 0.541–0.678; **Figure 3A–3C**).

There were significant differences among these 3 groups in terms of the 5-year OS (92.6%, 84.1%, and 65.2%; *P* < 0.001) (**Figure 3D**), PFS (83.9%, 76.4%, and 54.3%; *P* < 0.001) (**Figure 3E**), and DMFS (89.8%, 81.1%, and 68.9%; *P* < 0.001) (**Figure 3F**). Notably, HRG and MRG had a significantly worse OS (HRG *vs.* LGR, *P* < 0.001; MRG *vs.* LGR, *P* = 0.009) (**Figure 3D**), PFS (HRG *vs.* LGR, *P* < 0.001; MRG *vs.* LGR, *P* = 0.013) (**Figure 3E**), and DMFS (HRG *vs.* LGR, *P* < 0.001; MRG *vs.* LGR, *P* = 0.003) (**Figure 3F**) than the LRG. Kaplan-Meier analysis and the log-rank test also identified a significantly poorer OS (HRG *vs.* MGR, *P* = 0.014) (**Figure 3D**) and PFS (HRG *vs.* MGR, *P* = 0.006) (**Figure 3E**) among patients with a high ΔCONUT-EBV DNA score than among those with a medium ΔCONUT-EBV DNA score.

The results of our multivariate Cox analysis showed that the ΔCONUT-EBV DNA score was an independent prognostic factor for OS (HR: 1.652; 95% CI: 1.242–2.198; *P* = 0.001), PFS (HR: 1.772; 95% CI: 1.385–2.267; *P* < 0.001), and DMFS (HR: 1.874, 95% CI: 1.371–2.562; *P* < 0.001) in patients with advanced NPC who were treated with CCRT (**Table 5**).

**Table 5:** Univariate and multivariate analysis for 5-year OS, PFS, and DMFS combined with the novel prognosis predictor ΔCONUT score and EBV DNA

Endpoint	Variable	Univariate analysis		Multivariate analysis	
		HR (95% CI)	*P*	HR (95% CI)	*P*
OS	Age	1.681 (1.125–2.512)	0.011	1.564 (1.045–2.340)	0.030
	Gender	2.105 (1.214–3.648)	0.008	1.925 (1.107–3.347)	0.020
	UICC T stage	0.922 (0.578–1.471)	0.734	0.810 (0.472–1.390)	0.999
	UICC N stage	1.821 (1.219–2.720)	0.003	1.574 (1.046–2.368)	0.030
	Clinical stage	3.077 (0.975–9.712)	0.055	2.654 (0.731–9.638)	0.200
	ΔCONUT score	1.627 (1.045–2.535)	0.031	1.093 (0.475–2.514)	0.591
	EBV DNA	2.116 (1.396–3.207)	<0.001	0.884 (0.242–3.232)	0.634
	ΔCONUT-EBV DNA score	1.862 (1.415–2.450)	<0.001	1.652 (1.242–2.198)	0.001
PFS	Age	1.390 (0.976–1.979)	0.068	1.277 (0.894–1.826)	0.149
	Gender	1.357 (0.886–2.078)	0.161	1.262 (0.821–1.941)	0.299
	UICC T stage	0.933 (0.615–1.414)	0.743	0.799 (0.484–1.319)	0.635
	UICC N stage	1.499 (1.053–2.133)	0.025	1.178 (0.787–1.764)	0.130
	Clinical stage	1.955 (0.861–4.441)	0.109	1.903 (0.715–5.067)	0.196
	ΔCONUT score	1.417 (0.944–2.128)	0.093	0.736 (0.329–1.646)	0.922
	EBV DNA	1.931 (1.341–2.781)	<0.001	0.541 (0.166–1.766)	0.622
	ΔCONUT-EBV DNA score	1.772 (1.385–2.267)	<0.001	1.772 (1.385–2.267)	<0.001
DMFS	Age	1.031 (0.655–1.624)	0.894	0.928 (0.587–1.466)	0.847
	Gender	1.639 (0.917–2.930)	0.095	1.524 (0.849–2.737)	0.173
	UICC T stage	1.076 (0.619–1.869)	0.796	1.056 (0.555–2.007)	0.836
	UICC N stage	1.819 (1.149–2.881)	0.011	1.497 (0.897–2.497)	0.054
	Clinical stage	2.321 (0.731–7.366)	0.153	1.613 (0.419–6.218)	0.229
	ΔCONUT score	1.314 (0.774–2.233)	0.312	0.805 (0.275–2.357)	0.479
	EBV DNA	2.376 (1.462–3.860)	<0.001	0.973 (0.204–4.645)	0.523
	ΔCONUT-EBV DNA score	1.874 (1.371–2.562)	<0.001	1.874 (1.371–2.562)	<0.001

OS, overall survival; HR, hazard ratio; CI, confidence interval; N stage, node stage; T stage, tumor stage; UICC, Union for International Cancer Control; CONUT score, controlling nutritional status score; EBV DNA, Epstein-Barr virus deoxyribonucleic acid; PFS, progression-free survival; DMFS, distant metastasis-free survival.

## Discussion

Studies have recently shown that the CONUT score is a prognostic predictor for survival prognosis in many cancer types^11–16^. Moreover, the point at which the nutritional score should be measured during anti-cancer treatment remains unclear. In the present study, the prognostic values of the pre-CONUT, post-CONUT, and ΔCONUT scores were evaluated and compared in 433 patients with advanced NPC. The ΔCONUT score was found to be superior to the pre-CONUT and post-CONUT scores in terms of the predictive ability for prognosis, and it was an independent prognostic factor for OS. Moreover, our study suggested that a combination of the ΔCONUT score and the EBV DNA level was a novel tool for the prediction of poor future outcomes in patients with NPC.

Many reports have suggested that the CONUT score is a useful and convenient biomarker for estimating the nutritional status and predicting prognoses among patients with non-metastatic renal cell carcinoma^12^, esophageal cancer^13,14^, pancreatic ductal adenocarcinoma^15^, and stage 2/3 gastric cancer^15^. The CONUT score, an immunological and nutritional score, is calculated from the total lymphocyte count and total cholesterol and serum albumin levels^17^. Lymphocytes were found to be associated with cellular immunity against malignant cells^28,29^. Cholesterol reportedly reflects the nutritional status and cancer malignancy status^30,31^. Serum albumin level is an indicator of nutritional status^32^; however, it is more widely recognized as a marker of inflammation^33,34^. Previous studies have shown that low lymphocyte counts and low cholesterol and serum albumin levels are associated with poor prognoses in different cancers^35–39^.

This study is the first to assess the influence of changes in the CONUT score during treatment, so the ΔCONUT-EBV DNA score has potential applications in the development of nutritional and individualized treatments for the prognoses of patients with advanced NPC. In the present study, the ΔCONUT score was found to be superior to the pre-CONUT and post-CONUT scores in predicting survival for patients with NPC.

The mechanisms explaining why a high ΔCONUT score is associated with worse OS are not fully known. Side effects of anti-cancer treatment affect the nutritional status among patients with head and neck cancers. Oral mucositis is one of the most common side effects of anti-cancer treatments such as radiation therapy, chemotherapy, and immunotherapy^40,41^. It occurs in almost all patients receiving radiation therapy for head and neck cancers^42–44^. Clinically, its symptoms include anorexia, malnutrition (significant weight loss), and systemic infections. It also can influence treatment efficacy by causing interruptions in treatment or by resulting in dose reductions in chemotherapy and radiotherapy^45^. In turn, dose modification and treatment interruption have been associated with decreased survival^46–48^. Taken together, the ΔCONUT score might predict the prognoses of patients with NPC based on a combination of host malnutrition and immunity.

As mentioned in the Introduction, nutritional scores only reflect 1 aspect of the nutritional status and ignore tumor-related factors. In combination with nasopharyngeal and neck magnetic resonance imaging, nasopharyngoscopy, plasma EBV DNA levels and other blood indicators are routinely assessed in most hospitals. Across numerous studies, plasma EBV DNA levels have been shown to be associated with NPC stage^49–51^, suggesting a reliable and direct correlation between tumor burden and plasma EBV DNA levels^52^. The EBV DNA level is likely a reliable prognostic predictor and tumor-related factor for NPC^3,53,54^. The association between survival outcomes and the plasma EBV DNA levels have been investigated in many studies^20,24,25^. The cut-off values of plasma EBV DNA levels varied across these studies, and an optimal cut-off value of this liquid biopsy marker is still being determined^27,55^. Measurement of plasma EBV DNA levels is commonly used to diagnose NPC and to evaluate anti-cancer treatment and prognosis. Studies support a positive relationship between the EBV DNA level and tumor burden^24,25^. Finally, we further determined the complementary role of the ΔCONUT score in combination with pretreatment plasma EBV DNA.

In the subgroup analysis, the ΔCONUT score was found to be correlated with the OS of patients with a higher EBV DNA level, but not with the OS of patients with a lower EBV DNA level. Thus, the ΔCONUT-EBV DNA score, a novel combination prognostic marker, was introduced to improve the predictive value of the ΔCONUT score among patients with advanced NPC. Compared to the ΔCONUT score or pretreatment plasma EBV DNA level alone, the ΔCONUT-EBV DNA score was more predictive of the OS, PFS, and DMFS for patients with advanced NPC. Thus, patients with NPC who have a medium or high CONUT-EBV DNA score could benefit from a more intensive follow-up, even after curative CCRT with a nutritional intervention during treatment; this may be clinically beneficial in improving the treatment outcomes of these patients.

Nutritional and inflammation status have significant effects on the prognoses of cancer patients. Many studies have already shown that inflammatory markers, nutritional indices, or inflammation-based prognostic scores including red blood cell^56^, total lymphocyte count^57^, albumin^58–60^, hemoglobin^61,62^, serum pre-albumin^63,64^, transferrin^65^, serum C-reactive protein^66^, BMI^7,67–69^ and prognostic nutritional index (PNI)^70^ are closely associated with treatment outcomes in patients with NPC. Using these blood indicators could only reflect 1 aspect of the nutritional status, so the sensitivity of the scores may differ. These indicators could be easily affected by metabolism, food, and disease status^71–74^. The utility of BMI and PNI are useful to assess the nutritional status in clinical practice. Individual nutritional factors, such as hemoglobin, albumin, and BMI were not prognostic factors in Wang’s cohort of patients with NPC^75^. However, BMI and PNI are easily influenced by gender, age, and disease status. Using only BMI, PNI or 1 blood indicator to assess the nutritional status of patients may therefore introduce errors^76^. These nutritional or immune-nutritional scores are not always reliable in predicting the outcomes of cancers because they lack tumor-related factors. In addition, none of these scores are designed specifically for NPC patients.

However, the tumor-related factors, such as TNM stage and plasma EBV DNA levels only reflect 1 aspect of the tumor-related status. These factors are always reliable in predicting the treatment outcomes of cancers. The EBV DNA level is likely a useful prognostic predictor and tumor-related factor for NPC. The association between survival outcomes and plasma EBV DNA levels have been investigated in many studies^20,24,25^. The cut-off values of the plasma EBV DNA level varied across these studies, and an optimal cut-off value of this liquid biopsy marker is still being determined^27,55^.

The relationship between survival outcomes and nutritional or tumor-related factors needs to be further established in NPC patients, although both nutritional scores and plasma EBV DNA levels are used in the clinic. However, there are only a few clinical studies that have characterized the applicability of different nutritional scores when combined with tumor-related factors and the plasma EBV DNA levels in patients with NPC. The ΔCONUT-EBV DNA score includes plasma EBV DNA level and also assesses the change of immune-nutritional status during anti-cancer treatment, resulting in a particularly relevant multidimensional score. The novel combined ΔCONUT-EBV DNA scoring system provides more comprehensive prognostic information than individual nutritional indexes.

Our study had some limitations. First, it was a retrospective study performed at a single center. Second, other nutritional scores, such as PNI, BMI, and platelet:lymphocyte ratio, were not assessed. Thus, we do not know if changes in other nutritional scores during the treatment influenced the survival of patients with advanced NPC. Third, information on the side effects of anti-cancer treatment and food intake during the treatment was insufficient for the further analysis of OS, PFS, and DMFS. Thus, further prospective studies are required to establish the value of the ΔCONUT-EBV DNA score as a biomarker of prognosis and treatment outcomes in NPC.

## Conclusions

Taken together, the ΔCONUT-EBV DNA score, which is based on the ΔCONUT score and pretreatment plasma EBV DNA, has been integrated with the nutritional score and tumor-related factors, which may have more predictive information when compared to only 1 parameter. This score could be important and useful in clinical practice as a convenient, inexpensive biomarker for predicting outcomes for patients with advanced NPC treated with CCRT. As a novel and convenient biomarker, the ΔCONUT-EBV DNA score can be used in the development of nutritional and individualized treatments.
