# Supplementary material for: Prognostic significance of a combined and controlled nutritional status score and EBV-DNA in patients with advanced nasopharyngeal carcinoma: a long-term follow-up study
Source: Cancer Biol Med. 2021 Jun 16;19(4):551–64. doi: 10.20892/j.issn.2095-3941.2020.0627 (PMC9088186; doi:10.20892/j.issn.2095-3941.2020.0627)
Supplement: Supplementary file 1 [file cbm-19-551-s001.pdf]

Supplementary materials

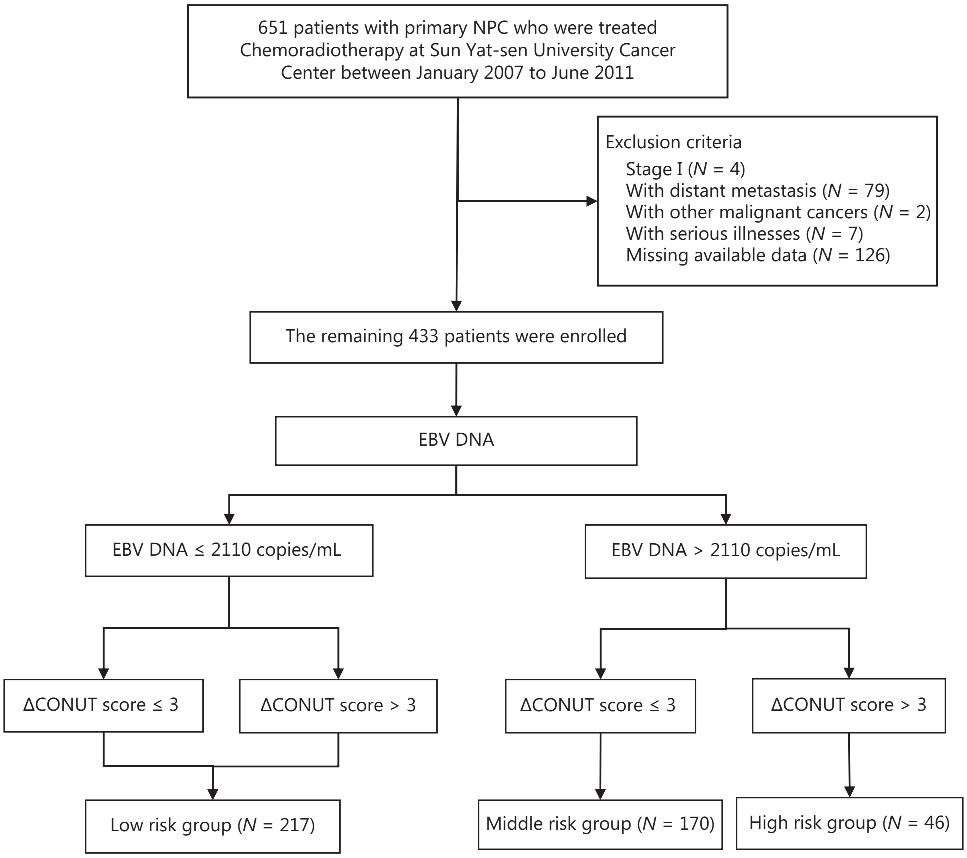

Figure S1 Flowchart showing the patient selection and classification.

Table S1 Nutritional status assessment according to the CONUT scoring system

Parameters	Malnutrition status			
	Normal	Light	Moderate	Severe
Total lymphocyte count (/mm^3)	≥1,600	1,200–1,599	800–1,199	<800
Score	0	1	2	3
Total cholesterol (mg/dL)	≥180	140–179	100–139	<100
Score	0	1	2	3
Serum albumin (g/dL)	≥3.50	3.00–3.49	2.50–2.99	<2.50
Score	0	2	4	6
Total score	0–1	2–4	5–8	9–12

CONUT, controlling nutritional status score.
